# Methadone Shortage in Morocco: A Challenge for Addiction Specialists

**DOI:** 10.1192/j.eurpsy.2026.11783

**Published:** 2026-07-31

**Authors:** I. Bouamoud, N. Barkallil, Z. Bencharfa, F. El Omari

**Affiliations:** Addictology, Arrazi Hospital, salé, Morocco

## Abstract

**Introduction:**

Methadone plays a central role in the treatment of opioid use disorders. Its recent shortage in Morocco has forced addiction centers to adapt rapidly, disrupting the working conditions of healthcare professionals.

**Objectives:**

This study analyzes the organizational, emotional, and perceptual impact of the methadone shortage on professionals, while identifying the challenges encountered and potential strategies to better manage future supply disruptions.

**Methods:**

A descriptive and analytical cross-sectional study was conducted among professionals from eight addiction centers using a structured questionnaire.

**Results:**

Professionals faced work overload, increased tensions with patients, and a lack of clear guidelines. Institutional communication was deemed insufficient, compelling teams to improvise solutions. Perceptions of methadone evolved: although considered useful, the strong dependence revealed by the abrupt cessation highlighted the need to expand therapeutic options.

**Conclusions:**

This crisis exposed organizational and institutional support weaknesses. It underscores the urgent need for a national shortage management plan, improved communication among stakeholders, and enhanced support for healthcare providers.

**Disclosure of Interest:**

None Declared

